# Mechanisms of Vasculogenic Mimicry in Ovarian Cancer

**DOI:** 10.3389/fonc.2019.00998

**Published:** 2019-09-27

**Authors:** Lízbeth Ayala-Domínguez, Leslie Olmedo-Nieva, J. Omar Muñoz-Bello, Adriana Contreras-Paredes, Joaquín Manzo-Merino, Imelda Martínez-Ramírez, Marcela Lizano

**Affiliations:** ^1^Programa de Doctorado en Ciencias Biomédicas, Universidad Nacional Autónoma de México, Mexico City, Mexico; ^2^Unidad de Investigación Biomédica en Cáncer, Instituto Nacional de Cancerología-Instituto de Investigaciones Biomédicas, Universidad Nacional Autónoma de México, Mexico City, Mexico; ^3^Programa de Doctorado en Ciencias Bioquímicas, Universidad Nacional Autónoma de México, Mexico City, Mexico; ^4^Cátedras CONACyT-Instituto Nacional de Cancerología, Mexico City, Mexico; ^5^Departamento de Medicina Genómica y Toxicología Ambiental, Instituto de Investigaciones Biomédicas, Universidad Nacional Autónoma de México, Mexico City, Mexico

**Keywords:** vasculogenic mimicry, ovarian cancer, signaling molecules, angiogenesis, anti-angiogenic therapy

## Abstract

Solid tumors carry out the formation of new vessels providing blood supply for growth, tumor maintenance, and metastasis. Several processes take place during tumor vascularization. In angiogenesis, new vessels are derived from endothelial cells of pre-existing vessels; while in vasculogenesis, new vessels are formed *de novo* from endothelial progenitor cells, creating an abnormal, immature, and disorganized vascular network. Moreover, highly aggressive tumor cells form structures similar to vessels, providing a pathway for perfusion; this process is named vasculogenic mimicry (VM), where vessel-like channels mimic the function of vessels and transport plasma and blood cells. VM is developed by numerous types of aggressive tumors, including ovarian carcinoma which is the second most common cause of death among gynecological cancers. VM has been associated with poor patient outcome and survival in ovarian cancer, although the involved mechanisms are still under investigation. Several signaling molecules have an important role in VM in ovarian cancer, by regulating the expression of genes related to vascular, embryogenic, and hypoxic signaling pathways. In this review, we provide an overview of the current knowledge of the signaling molecules involved in the promotion and regulation of VM in ovarian cancer. The clinical implications and the potential benefit of identification and targeting of VM related molecules for ovarian cancer treatment are also discussed.

## Introduction

Ovarian cancer is the second most common and lethal gynecological cancer (1). Among ovarian cancer types, the epithelial ovarian cancer accounts for almost 90% of such malignancy (2), which is usually diagnosed in advanced aggressive stages due to its asymptomatic nature. Extensive tumor invasion, peritoneal metastases, and treatment failure are frequent in advanced epithelial ovarian cancer (3).

The normal physiology of the ovary is characterized by increased permeability of blood vessels during follicular development, ovulation, and subsequent formation of the corpus luteum, with cyclic changes in the formation, differentiation, and regression of ovarian vasculature (4). These vascular processes are deregulated in ovarian cancer, which is characterized by intense neovascularization (5, 6). Neovasculature in ovarian cancer is formed not only from endothelial cells but also from endothelial progenitor cells and/or cells from the tumor itself, allowing the supply of blood and nutrients to the tumor with great efficiency (7).

The versatility of the vascularization processes in ovarian cancer could partially explain its aggressive nature and the limited efficacy of anti-angiogenic therapies (8). An alternative vascularization process, vasculogenic mimicry (VM), has been shown to increase after anti-angiogenic treatment with bevacizumab, in preclinical models of ovarian cancer (9). This finding suggests that VM could be a strategy for escaping anti-angiogenic treatment, highlighting the importance to study the mechanisms involved in vascular remodeling.

In this review, we provide an overview of the current knowledge of the mechanisms and signaling molecules involved in the promotion and regulation of VM in ovarian cancer, its clinical implications and the potential benefit of therapeutic approaches based on the identification and targeting of VM related molecules.

## Tumor Vascularization Processes in Ovarian Cancer

The study of the vascularization processes in solid tumors has gained importance due to its implication in growth and metastasis, as well as its possible implication for anti-angiogenic treatment resistance (10). The most studied tumor vascularization process is angiogenesis, although tumor tissue has the capacity to generate its own vasculature from alternative mechanisms such as vasculogenesis, vessel co-option, and VM (11–13).

### Angiogenesis

Angiogenesis is a highly regulated process aimed to produce new blood vessels with a key role in development and postnatal life; it is also involved in invasion, growth, and metastasis of solid tumors (14, 15). The onset of angiogenesis occurs in response to hypoxia or ischemia where pro-angiogenic signals overcome anti-angiogenic signals. The vascular endothelial growth factor A (VEGF-A) is the master regulator of angiogenesis, both in physiological and pathological conditions (16). During angiogenesis activation, a complex signaling cascade begins, leading to the proliferation of endothelial cells (ECs) that assemble new vascular networks from the pre-existing vessels, increasing permeability and leakage, and restoring the supply of oxygen and nutrients toward the tumor mass (15, 17).

Angiogenesis is essential for the growth of ovarian cancer cells and their spreading to the peritoneum. VEGF-A has been associated with peritoneal ECs proliferation, migration, and formation of tube-like structures (18). The inhibition of VEGF-A does not revert these processes, suggesting that another pro-angiogenic factors secreted by surrounding ovarian cancer cells or their microenvironment could be involved in the angiogenic activation of peritoneal ECs during metastasis (18, 19). A high level of pro-angiogenic signals has been associated with the formation of ascites, a frequent feature of advanced ovarian cancer (20, 21).

### Vasculogenesis

Vasculogenesis, a *de novo* vessel formation process, is distinguished by the *in situ* differentiation of ECs from myeloid cells or endothelial progenitor cells (EPCs). This process takes place at the beginning of vascular development and during post-natal life (11, 22). Myeloid cells and EPCs are recruited by pro-angiogenic or pro-inflammatory factors to the tumor vascular bed, where they differentiate into ECs and give place to neovasculature (23–25). Vasculogenesis has a modest impact on tumor vascularization when the angiogenesis pathway is active, however, it is recognized as an important rescue process when this pathway is blocked (10, 26). For instance, when angiogenesis is inhibited after anti-angiogenic treatment or radiotherapy, myeloid cells, and EPCs are recruited by the stroma-derived factor 1 (SDF-1) in response to an increased level of hypoxia-inducible factor 1α (HIF-1α) (10, 26).

Vasculogenesis has an important role in ovarian cancer. It has been related to treatment resistance as a consequence of the overexpression of matrix metalloproteinase 2 and 9 (MMP-2 and MMP-9) after radiotherapy (27). Furthermore, CD34+ EPCs from peripheral blood incorporate into vasculogenic active sites (25) as well as CD11b+ and CD11c+ myeloid cells, recruited by SDF-1 and β-defensins, that contribute to vasculogenesis (28). β-defensins chemoattract CD11c+ dendritic cell precursors and then VEGF-A induces endothelial-like specialization mediated by VEGF receptor 2 (VEGFR-2); interestingly, recruitment of CD11c+ cells has also been found in ascites (28).

### Vessel Co-option

Vessel co-option is a process that differs from angiogenesis; instead of inducing the proliferation of ECs, tumor cells grow by adhering to nearby blood vessels (15). Different patterns of vessel co-option have been described in brain, lung, and liver cancers (12). In glioblastoma, CDC42+ CD44+ tumor cells migrate toward a blood vessel in response to a bradykinin gradient created by ECs; when these cells reach the vessel, they fuse with the pericytes or adhere to the basement membrane (12). Vessel co-option has been observed in a mouse model of ovarian cancer (29), where endostatin inhibited vessel co-option by blocking the attachment of ovarian cancer cells to peritoneal vessels through integrins α_5_β_1_.

It has been proposed that after the tumor grows by vessel co-option, co-opted vessels regress, and the tumor enters into an avascular phase followed by the induction of peritumoral angiogenesis (30). Vessel co-option facilitates the metastasis of tumor cells since it increases their motility and migration. There is evidence that tumors can switch between angiogenic and non-angiogenic growth during progression and that they can contain angiogenic and non-angiogenic areas (12). The association between vessel co-option and resistance to anti-angiogenic treatment is not clear, since vessel co-option could be one cause of the resistance to anti-angiogenic treatment or it could be a consequence of the aggressive nature of cancer cells in response to anti-angiogenic treatment (10, 31).

### Vasculogenic Mimicry

VM is a process by which tumor cells form capillary-like structures, mimicking the embryonic vascular network pattern, without inducing the proliferation of ECs (15). This process increases blood perfusion, allows tumor cells to obtain oxygen and nutrients, and promotes cancer progression (13, 32). It has been proposed that VM is carried out through cancer stem cell (CSC) trans-differentiation into endothelial-like cells (13, 33). Moreover, tumor cells involved in VM resemble mesenchymal cells derived from epithelial to mesenchymal transition (EMT), which is characterized by a down-regulation of epithelial markers (cytokeratin, for example), a loss of cell polarity (E-cadherin, occludin), and the upregulation of mesenchymal markers (vimentin, N-cadherin, fibronectin) (34, 35). Furthermore, these VM cells have an endothelial phenotype. VM has been associated with unfavorable outcome in patients with malignant tumors (36) and has an important participation in tumor invasion and metastasis (37).

Cell-lined vasculature compatible with VM has been observed in ~30–37% of ovarian cancers (38, 39). The presence of such cell-lined vasculature was associated with a higher histological grade and more aggressive tumors. An increased number of VM channels were found in poorly differentiated ovarian cancer cells (40). The presence of VM, combined with the expression of CD133, was positively associated with poor prognosis in patients with ovarian cancer (41). In a preclinical model of ovarian cancer, an accelerated metastasis was observed together with hypoxia and VM after anti-angiogenic treatment with bevacizumab (an anti-VEGF-A monoclonal antibody) (9). All these findings highlight the importance of identifying the underlying mechanisms and the signaling molecules involved in VM to evaluate their prognostic or predictive value, as well as their use as potential targets for developing more effective therapies (42).

## Structural and Functional Description of VM

In 1999, Maniotis et al. performed *in vitro* and *in vivo* assays in melanoma and found two VM types: a tubular type, and a patterned matrix type (13). The tubular type consists of hollow cords formed by tumor cells that give place to a tubular network. These tubular structures are also connected to other channels that contain red blood cells. Further studies showed that in some cases, a mixture of tumor cells and ECs form those tubular structures (43). The patterned matrix type consists of a network of loops formed by matrix layers that surround clusters of tumor cells. These layers are not uniformly spaced; therefore, the transport of fluid is not uniform around the cell cluster. However, this patterned matrix could provide a greater surface area for diffusion than that provided by a tubular structure (44). The reorganization of tumor cells into cords or clusters, as well as the formation of matrix layers involve mechanisms such as cell-cell adhesion, migration, and extracellular matrix remodeling, where several signaling molecules have been associated with VM.

VM structures have been identified in tissue samples as positive for periodic acid-Schiff (PAS) and negative for EC markers such as CD31 or CD34 (42). PAS+ regions are rich in components of the extracellular matrix, like laminin. Recently, it has been shown that PAS+ regions could also represent non-functional structures unrelated to VM in *in vitro* studies (45). Moreover, the different vascular structures aimed to conduct fluids within the tumor share several features. Thus, in order to identify the structures that truly correspond to VM as well as to distinguish them from similar structures from the other vascularization processes, it is necessary to assess their architectural and functional features, in addition to their composition. Recently, Valdivia et al. (46) described the architectural and functional features required for differentiating VM from other vascular structures in tumors (46). Whilst blood and lymph vessels are formed by a single line of ECs surrounded by a continuous and non-continuous basement membrane, respectively, VM structures are formed by cancer cells resting on an inner glycoprotein rich matrix (46). The authors propose that, in addition to the traditional architectural features to identify a VM structure (PAS+ and without EC markers), the presence of red blood cells within the lumen of the structure is an indicator of VM functionality (46).

Early studies in breast cancer and melanoma have shown that tubular and patterned matrix VM structures are capable of conducting plasma and red blood cells *in vitro* and *in vivo* (44). Maniotis et al. (47) showed that VM structures formed by aggressive melanoma cells *in vitro* conducted a tracer by direct microinjection and passive diffusion (47); moreover, the matrix pattern also contained red blood cells. Shirakawa et al. (48) used two breast cancer mice models to evaluate tumor blood flow with micro-magnetic resonance angiography imaging (48). They found that aggressive tumor cells formed VM structures in the center of the tumor, while non-aggressive cells showed necrotic cores. Angiogenic vessels were present in tumor periphery in both types of tumors and blood flow was higher in VM structures than in necrotic cores. Clarijs et al. (49) used a tracer to study perfusion in a melanoma mouse model (49). Tracer distribution suggested that blood vessels could be in contact with VM structures, allowing the perfusion of the latter, mediated by at least three mechanisms: the anastomosis of VM structures to blood vessels (50), an increased leakage from blood vessels (47, 51), and through anticoagulant control exerted by aggressive tumor cells (50).

## Mechanisms and Signaling Molecules Involved in VM in Ovarian Cancer

**Figure 1:**
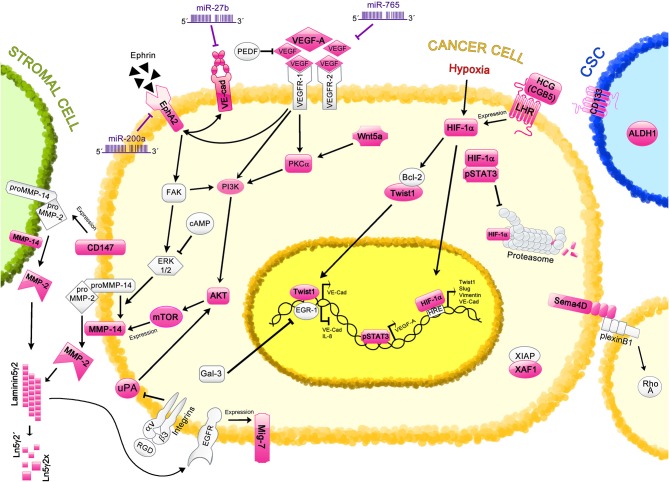
Regulation of Vasculogenic Mimicry by cell signaling molecules in ovarian cancer. Different proteins and signaling pathways involved in VM are shown; those characterized in ovarian cancer VM are highlighted in pink. Cancer cells are depicted in yellow; stromal cell, in green; and cancer stem cell (CSC), in blue. The VE-cadherin/EphA2/MMP-14/MMP-2/Ln5γ2 axis is the main mechanism involved in the induction of VM. This axis is regulated by miR-27b and miR-200a. Other proteins such as VEGF-A (regulated by miR-765 and pSTAT3), CD147, uPA, and Twist1 also regulate this axis through different pathways. Hypoxic-related protein HIF-1α induces the expression of Twist1, VE-Cadherin (VE-Cad), Slug and Vimentin, which are involved in VM induction; moreover, proteins such as pSTAT3, HCG, and LHR regulate the levels of HIF-1α. CSC markers, including ALDH and CD133 are found in ovarian tissues with VM structures. Different cell signaling pathways are also involved in VM, such as Wnt5a, and RTKs pathways, which strongly correlate with VM formation. Additional molecules that have been proposed in VM regulation in ovarian cancer are Sema4, XAF1, and Mig-7, however the precise mechanisms remain unclear.

Several mechanisms are involved in VM, including those related to the capacity of aggressive tumor cells to resemble features of the ECs such as cell adhesion (52), migration (53), extracellular matrix remodeling (54), perfusion (50), and maturation of blood vessels (55). Moreover, CSCs promote VM by deregulating pathways involved in embryonic development, such as the transforming growth factor β (TGF-β) (56–58), Wnt (59), Notch (60, 61), Nodal (62–64), and the Hippo pathways (65–67), among others. EMT also plays an important role in VM, and encompasses the pathways previously mentioned as well as transcription factors such as Twist1/2 (68), Snail/Slug (69), and ZEB1/2 (34). Moreover, signaling molecules related to hypoxia (70), inflammation (71–75), and metabolism (76–79) also have an impact on VM. The novel findings regarding these mechanisms and their signaling molecules in the regulation of VM in ovarian cancer are presented in this section and are summarized in Figure 1. Additional proteins and signaling pathways identified in other cancer types are shown in Table 1.

**Table 1:** Signaling molecules and mechanisms that regulate VM in several types of cancer.

**Signaling molecules**	**Function in VM**	**Cancer type**	**References**
TF/TFPI-1,−2	Enhances perfusion in VM structures by the anticoagulant activity of TFPI-1. TFPI-2 contributes to the activation of MMP-2.	Melanoma	(50)
PDGF-B/PDGFR-β	PDGF-B recruits pericytes to support the maturation and stabilization of vascular networks.	Melanoma	(55)
TGF-β	Induces EMT and upregulates the expression of MMP-14 and MMP-2	Melanoma Breast cancer	(56–58)
Nodal	Maintains the transdifferentiated phenotype, increases the expression of VE-cadherin, and promotes EMT	Melanoma Breast cancer	(62–64)
Notch	Promotes CSCs renewal and upregulates the expression of MMP-2 through the activation of the VEGF/VEGFR-1 pathway	Melanoma	(60, 61)
Hippo (YAP/Sox2/Oct4)	Promotes CSCs renewal	Lung cancer	(65–67)
HIF1α/Bcl2/Twist1	Induces EMT	Hepatocellular	(68)
ZEB1/2	Induces EMT and upregulates the expression of VE-cadherin, VEGFR-1, and MMPs	Hepatocellular	(34)
HIF1α/Bcl2	Upregulates the expression of VE-cadherin	Melanoma	(70)
IL-8/CXCR1, 2	Upregulates the expression of MMP-2	Melanoma Glioblastoma Breast cancer	(71–74)
Gal-3	Upregulates the expression of VE-cadherin and IL-8 by preventing the binding of the transcriptional repressor EGR-1	Melanoma	(75)
cAMP	Inhibits VM by activating Epac/Rap1 or by inactivating PI3K pathway through ERK1/2 inhibition	Melanoma	(76)
DDAH/NO	Induces VM by unknown mechanism	Breast cancer	(77)
COX2/EP3	Increases the activity of MMP-2	Breast cancer	(78, 79)

*VM, vasculogenic mimicry; EMT, epithelial to mesenchymal transition; CSC, cancer stem cell*.

Vascular endothelial (VE)-cadherin, one of the main participants in cell-cell adhesion in endothelial cells, is strongly associated with VM formation (80). This protein recruits the EC-related kinase Ephrin-A2 receptor (EphA2) to the cell membrane (52), increasing the phosphorylation of the focal adhesion kinase (FAK). Consequently, the activation of extracellular regulatory kinases 1 and 2 (ERK1/2) signaling pathway is promoted, allowing the activation of MMP-14 (81). Then, MMP-14 converts proMMP-2 into active MMP-2. These MMPs degrade extracellular matrix components and facilitate invasion, metastasis, and VM (82). Particularly, MMP-2 and MMP-14 induce the Laminin5γ2 (Ln5γ2) cleavage (53, 83). Although the precise mechanism has not been clearly described, it is known that MMP-2 cleavages Ln5γ2 into two pro-metastatic fragments (Ln5γ2′ and Ln5γ2x) (53). Together, these results indicate that the VE-cadherin/EphA2/MMP-2/Ln5γ2 axis is the main regulator of VM.

Interestingly, high expression of VE-cadherin and EphA2 has been found in clinical samples from ovarian cancer patients that exhibit a highly invasive phenotype (84, 85). Additionally, other studies demonstrated that MMP-2 and MMP-14 are also overexpressed in ovarian cancer samples, which is associated with poor clinical outcome (38, 86). It is worth to mention that those findings strongly correlated with the presence of VM structures, suggesting that these molecules are important players in this process.

The Phosphatidylinositol 3-kinase (PI3K) cell signaling pathway regulates MMPs expression in VM (87). This pathway is activated through FAK phosphorylation (88), impacting in the expression of MMP-14. Moreover, the PI3K pathway is frequently activated in ovarian cancer, probably impacting VM (89).

Another regulator of VM is the urokinase plasminogen activator (uPA), which is required to induce the degradation of the extracellular matrix, impacting in tumor angiogenesis. The overexpression of uPA positively correlates with VM formation in ovarian cancer tissues (54). In addition, it was demonstrated that in SKOV-3 and OVCAR-3 ovarian cancer cells, the ablation of uPA expression results in a decrement of complete VM structures formation and such mechanism involves the participation of AKT/mTOR/MMP-2/Laminin5γ2 signal pathways (54).

VEGF-A also upregulates the expression MMPs. It has been shown that in melanoma, VEGF-A induces VM formation by activating the PI3K/protein kinase C α (PKCα) pathway via VEGFR-1 signaling (90). However, in glioblastoma, VM is induced by the VEGFR-2 signaling (91). In an *in vitro* model of ovarian cancer using SKOV-3 and OVCAR-3 cells, VEGF-A promoted migration, invasion, and VM by up-regulating MMPs via EphA2 (92). This suggests that VEGF-A interacts with the VE-cadherin/EphA2/MMP-2/Ln5γ2 axis in the regulation of VM in ovarian cancer.

The plasma membrane glycoprotein CD147 plays an important role during tumor progression, invasion and metastasis, regulating metalloproteinases expression in peritumoral stromal cells. Invasion capability was evaluated in two different cell lines derived from ovarian cancer with different invasion activity: CABA I and SKOV3 (93). A correlation of CD147 expression with tumor invasiveness, protease activity (MMP-2 and MMP-9), and vascular channels formation was observed. Interestingly, when high invasive cell line was treated with small interfering RNA against CD147, a suppression of non-EC-lined channels was observed. In addition, when CD147 was overexpressed in a low invasive cell line, those cells exhibited an increase of tumor invasion and vascular channel formation. These data suggest that CD147 plays an important role in VM induction in ovarian tumors and CD147 could be an attractive target for therapeutic intervention (93).

Furthermore, Ln5γ2 activates the endothelial growth factor receptor (EGFR) which promotes the expression of the migration-inducing protein 7 (Mig-7), stimulating invasion and VM (94). A study carried out in ovarian cancer samples revealed an association of VM with VE-cadherin and Mig-7 expression (84). It was observed that ovarian tumors without VM frequently expressed low levels of VE-cadherin compared to those with VM. Meanwhile, Mig-7 expression was increased in tumor samples compared to normal tissues, positively correlating with VM and VE-cadherin expression (84).

Some elements involved in apoptosis have been associated with the formation of VM structures, such as the pro-apoptotic XIAP-associated factor 1 (XAF1). Recently, *in vivo* xenograft models of ovarian cancer have shown that the overexpression of XAF1 decreases the number of VM structures (39). Moreover, *in vitro* assays with SKOV3 cells revealed that proliferation, migration and invasion were inhibited, and the levels of VEGF were reduced when XAF1 was exogenously overexpressed (39). Therefore, XAF1 is a potent negative regulator of VM in ovarian cancer.

It has been shown that VEGF-A regulates the expression of the axon guidance factor semaphorin 4D (Sema4D) (95), which has been identified as a promotor of VM in non-small cell lung cancer (96), where the recognition of Sema4D by the plexin B1 receptor activates the small GTPase RhoA, which is implicated cell motility. However, when plexin B1 was inhibited, a disruption of the RhoA/ROCK signaling occurred, suppressing VM formation. Additionally, the presence of VM in clinical specimens correlated with increased levels of Sema4D (96). In an ovarian cancer cell line (A2780), soluble Sema4D promoted angiogenesis and VM via plexin B1 (95); moreover, in clinical samples from patients, a high expression of Sema4D had a positive correlation with the malignant degree of epithelial ovarian cancer. Interestingly, it was observed that VEGFR-2, plexin-B1, and Sema4D control the expression of CD31, MMP-2, and VE-cadherin in ovarian cancer cells, which are the markers and initiators of angiogenesis and VM (95).

CSCs are present in ovarian cancer and are positive for CD133, a unique surface marker of CSCs (97). It is known that CD133+ cells promote VM in several cancer types (41, 91, 98–101). The combined expression of CD133 and VM in samples from patients was associated with high-grade ovarian carcinoma, late-stage disease, non-response to chemotherapy and shorter overall survival (41). The trans-differentiation of CD133+ CSCs into ECs may induce VM formation and the expression of EC markers such as VE-cadherin (101) and VEGFR-2 (91). Moreover, it has been shown that in hypoxic environment the subpopulation of CD133+ CSCs is augmented when Twist1 was overexpressed (100). This finding shows that hypoxia may exert an effect on CSCs that probably leads to VM formation.

CSCs can also exhibit a high activity of aldehyde dehydrogenase-1 (ALDH1) (97). The expression of ALDH1 has been evaluated in different types of tumor, including breast cancer, colorectal cancer, and ovarian cancer and strongly correlates with VM, determining an unfavorable clinical outcome (102–104). Although the precise mechanism has not been described, it is known that ALDH1 and VM increase in response to hypoxia (105).

Hypoxia regulates several pathways in cancer, such as angiogenesis, and it has been related to VM in melanoma, glioblastoma, ovarian cancer, and hepatocellular carcinoma (68–70, 106). Hypoxia induces VM formation by up-regulating VE-cadherin expression. The main effectors of this pathways, HIF-1α and HIF-2α, positively regulated VE-cadherin expression; this effect is through the binding of HIF to hypoxia response elements (HRE) located in VE-cadherin promoter in glioblastoma cells (106). Interestingly, it was observed that EMT is promoted in a hypoxic environment and as a result, VM was induced in SKOV3 and OVCAR3 cells (69). *In vitro* assays showed that hypoxia leads to increased invasion, migration and an enhancement of MMP-2 activity. Therefore, EMT induction as a response to hypoxia is a master regulator of VM in ovarian cancer cells. Moreover, this study demonstrated that in ovarian cancer samples, the levels of HIF-1α were strongly associated with VM formation and the expression of Twist1, Slug, and Vimentin.

Another important regulator of VM under hypoxic conditions are the signal transducer and activator of transcription 3 (STAT3) and the phospho-STAT3 (p-STAT3). It has been suggested that p-STAT3 promote VM, this is due to the binding of pSTAT3 to HIF-1α, which in turn delays its degradation (107, 108). In gastric adenocarcinoma, VM was associated with an increased expression of HIF-1α, STAT3, and p-STAT3 (109). Moreover, STAT3 acts as a transcription factor in VEGF-A transcription (110). Interestingly, in SKOV3 cells p-STAT3 was found in the nucleus, suggesting that was transcriptionally active (111). In addition, when STAT3 was inhibited, the formation of VM structures was completely avoided, suggesting that p-STAT3 is an important regulator of VM in ovarian cancer cells.

The Wnt family members regulate EC differentiation and vascular development (112) and has been associated with VM. In glioma and colon cancer, the canonical Wnt/β-catenin pathway induced VM by increasing the expression of VEGFR-2 and VE-cadherin (59, 113). Interestingly, in ovarian cancer, the non-canonical Wnt signaling is implicated in VM formation. It was found that Wnt5a is overexpressed in tumor samples and is associated with VM (114). Moreover, *in vitro* analysis revealed that Wnt5a overexpression is linked to PKC pathway activation. Furthermore, it was shown that Wnt5a overexpression induced EMT, increased invasion and motility of SKOV3 cells (114).

An important proangiogenic factor in ovary is the human gonadotropin (HCG). The fifth subunit of β-HCG, CGB5, was shown to promote VM formation *in vitro* in OVCAR3 cells (115). Additionally, overexpression of CGB5 induced the growth of ovarian cancer cells in a xenograft murine model, as well as VM (116). It was also shown that the activation of luteinizing hormone receptor (LHR), which is the HCG receptor, is required for the promotion of VM formation by CGB5. In another study, it was found that ovarian cancer cells exogenously expressing HCG induced an overexpression of HIF-1α. Importantly, vascular markers such as CD31 and VEGF were also upregulated in those cells (117). Therefore, the HCG/LHR axis induces VM by HIF-1α regulation in ovarian cancer.

## Micro-RNAs as Regulators of VM in Ovarian Cancer

Micro-RNAs (miRNAs) are single stranded and non-coding RNA molecules of 19-25 nucleotides in length that have a post-transcriptional regulatory function (118). Different studies have demonstrated that miRNAs are involved in several physiological processes such as cell proliferation, invasion, migration, differentiation, as well as pathological processes including angiogenesis and VM (119–122). The dysregulation in the expression of these RNA molecules is often observed in numerous types of cancer. Diverse studies demonstrate that miRNAs post-transcriptionally regulate different signaling molecules involved in VM process (123–136); examples of these miRNAs are enlisted in Table 2.

**Table 2:** VM related miRNAs in different types of cancer.

**miRNA**	**Type of cancer**	**miRNA target**	**References**
miR-26b	HCCramya Glioma	VE-cadherin, Snail and MMP-2ramya EphA2	(123, 124)
miR-141	Gliomaramya Renal carcinoma	EphA2	(122, 125)
miR-27a/b	Ovarian cancer HCC	VE-cadherinramya Twist1	(126, 127)
miR-101	HCC	TGF-βR, Smad2 and SDF1	(129)
miR-200a	Ovarian cancer	EphA2	(85)
miR-204	Breast cancer	PI3K, c-SRC	(130)
miR-373	Glioma	EGFR	(131)
miR-186	Gastric cancerramya Prostate cancer	Twist1	(132, 133)
miR-29b	HCC	STAT3 and MMP-2	(134)
miR-193b	Breast cancer	DDAHI	(77)
miR-539-5p	Glioma	Twist1	(135)
miR-490-3p	Breast cancer	Twist1	(136)
miR-765	Ovarian cancer	VEGF-A	(128)

*VM, vasculogenic mimicry; HCC, hepatocellular carcinoma*.

A well-described miRNA family is miR-26, which includes miR-26a and miR-26b. Those are commonly downregulated in several types of cancer such as glioma, HCC, and gastric cancer (124, 137, 138). For instance, in gastric cancer miR-26a and−26b suppress angiogenesis by targeting hormone growth factor (HGF) mRNA and consequently affecting HGF/VEGF signaling (138). Moreover, in HCC miR-26b has been identified as tumor suppressor since its down-regulation promotes VM and angiogenesis (123).

Similarly, another cluster of miRNAs belonging to the miR-200 family (miR-141, miR-200a, miR-200b, miR-200c, and miR-429) has been widely studied in several types of cancers (125, 139–142). It has been shown that miR-141 overexpression inhibits VM formation through directly targeting EphA2 transcript, decreasing EphA2 protein levels in glioma and renal carcinomas (122, 125).

Hitherto, three miRNAs (miR-200a, miR-27b, and miR-765) have been described as VM regulators in ovarian cancer through directly targeting 3′UTRs of VM-related transcripts (85, 126, 128) (Table 3). The miR-200a was the first microRNA found in ovarian cancer capable of regulating VM (85). Tumors with low miR-200a expression correlate with the presence of VM structures and poor overall survival. An inverse correlation between mRNA and protein EphA2 levels and miR-200a expression was observed in ovarian cancer samples, suggesting a direct regulation among them. *In silico* assays revealed a miR-200a binding site at EphA2 3′UTR; this observation was confirmed in SKOV3 ovarian cancer cells, where a direct binding of miR-200a to EphA2 3′UTR was observed through luciferase assays. Consequently, the levels of EphA2 protein and mRNA decreased in this model. In agreement, it was shown that the EphA2 overexpression restores VM in miR-200a expressing cells, indicating that miR-200a inhibits VM by mainly targeting EphA2 (85).

**Table 3:** VM related miRNAs in ovarian cancer.

**miRNA**	**Expression in OC (↑up ↓down)**	**Target related to VM in OC**	**Direct/indirect VM target**	**References**
**miRNAs RELATED WITH VM IN OVARIAN CANCER**				
miR-27b	↓down	VE-cadherin	Direct 3'UTR binding	(126)
miR-200a	↓down	EphA2	Direct 3'UTR binding	(85)
miR-765	↓down	VEGF-A	Direct 3'UTR binding	(128)
**miRNAs PROBABLY RELATED WITH VM IN OVARIAN CANCER**				
miR-92	↑up	HIF-1α	Indirectly by targeting HIF inhibitor VHL	(143)
miR-199a-5p	↓down in Hypoxic OC		Direct 3'UTR binding	(144)
miR-199	↓down		By protein levels	(145)
miR-125	↓down		By protein levels	(145)
miR-138	↓down in invasive OC		Direct 3'UTR binding	(146)
miR-145	↓down		Indirectly by targeting p70S6K1	(147)
miR-718	↓down	VEGF	Direct 3'UTR binding	(148)
miR-126	↓down		By protein levels	(149)
miR-497	↓down		Direct 3'UTR binding	(150)
miR-92	↑up		Indirectly by targeting HIF inhibitor VHL and increasing HIF-1α	(143)
miR-199	↓down		By mRNA levels	(145)
miR-125	↓down		By mRNA levels	(145)
miR-145	↓down		Indirectly by targeting p70S6K1	(147)
miR-520d-3p	↓down	EphA2	Direct 3'UTR binding	(151)
miR-365	↓down	Wnt5a	Direct 3'UTR binding	(152)
miR-490-3p	↓down	MMP-2	By mRNA and protein levels	(153)
miR-106b	↓down		By mRNA and protein levels	(154)
miR-122	↓down		By protein levels	(155)
miR-122	↓down	MMP-14	By protein levels	(155)
miR-15a-3p	↓down	Twist1	Direct 3'UTR binding	(156)
miR-532-5p	↓down		Direct 3'UTR binding	(157)
miR-219-5p	↓down		Direct 3'UTR binding	(158)
miR-320	↓down		Direct 3'UTR binding	(159)
miR-548c	↓down		Direct 3'UTR binding	(160)
miR-214	↓down	SEMA4D	Direct 3'UTR binding	(161)
miR-193b	↓down	uPA	Direct 3'UTR binding	(162)
Mir-23b	↓down		By mRNA and protein levels	(163)
miR-519a	↓down	STAT3	Direct 3'UTR binding	(164)

*VM, vasculogenic mimicry; OC, ovarian cancer*.

Previously, it has been described that VE-cadherin expression is related to VM formation in different types of cancer. A bioinformatic study identified miR-27b as putative regulator of VE-cadherin by the detection of a binding site at VE-cadherin 3′UTR. Concordantly with this result, luciferase assays demonstrated that miR-27b binds to VE-cadherin mRNA 3′UTR in ovarian cancer cells. Furthermore, expression levels of VE-cadherin mRNA and protein in different ovarian cancer cell lines negatively correlate with miR-27b expression. Low metastatic cell lines OVCAR3 and SKOV3 express high amounts of miR-27b and low VE-cadherin mRNA, compared to metastatic cells ES2 and Hey1B that exhibit low amounts of miR-27b and high VE-cadherin mRNA. Overexpression of miR-27b on high VE-cadherin expressing cells decreases VE-cadherin mRNA and protein levels. When miR-27b is overexpressed in metastatic ovarian cancer cell lines (Hey1B and ES2), the migration, invasion, and VM are decreased in *in vivo* models (126).

A recent study aimed to determine the set of miRNAs regulated in an early stage before complete VM establishment under hypoxia conditions. It was shown that SKOV3 ovarian cancer cells grown under hypoxia conditions form a higher number of 3D capillary-like structures than those cells grown under normoxia conditions (128). A set of miRNAs involved in the regulation of tumorigenesis-related pathways, as well as several genes involved in VM and angiogenesis was found. Among them, miR-765 was highly downregulated under hypoxia (128). Moreover, its restoration promotes a dramatic inhibition of 3D capillary-like structures and down-regulates VEGF-A expression. Importantly, it was demonstrated that VEGF-A mRNA is a direct target of miR-765, since it binds to VEGF-A 3'UTR. Additionally, low levels of miR-765 and high levels of VEGF-A were associated with low overall survival from a cohort of 1,485 ovarian cancer patients (128).

Although only three miRNAs have been directly associated with VM in ovarian cancer, several signaling pathways, and proteins controlling this mechanism are regulated by miRNAs (143–164); therefore, these non-coding transcripts could have a potential role on VM regulation. Table 3 shows the common VM targets in ovarian cancer that are regulated by miRNAs.

## Clinical Implications of the Signaling Molecules of VM in Ovarian Cancer

Anti-angiogenic therapies have shown limited effects against cancer progression, due to alternative vascularization processes, such as VM, triggered by aggressive tumor cells (10). The knowledge of the mechanisms and signaling molecules involved in VM may lead to the development of novel anti-vascularization therapies that overcome the limitations found in conventional therapies. Therefore, it is necessary to explore the possible therapeutical strategies that could improve the clinical outcome of ovarian cancer patients.

Therapies targeting VM have not been developed in ovarian cancer so far. However, some inhibitory molecules of VM elements have been studied and have shown promising anti-VM effects (165–174). These inhibitor molecules are summarized in Table 4.

**Table 4:** Inhibitor molecules that target VM-related proteins.

**VM-related protein**	**VM Inhibitor**	**Cancer type**	**Drug action**	**Reference**
CD133	3-phenylthiazolo [3,2-a] benzimidazoles (4b Compound)	Breast cancer Colon cancer	Inhibits cell surface expression of CD133.	(165)
Mig-7	D-39 (derived from medicinal plant *Liriope muscari*)	Ovarian cancer	Suppresses Mig-7 expression.	(166)
uPA	WX-671 (Mesupron or Upamostat)	Pancreatic cancer	Inhibits Serine proteases (including uPA).	(167)
XAF1	ATRA (All *trans* retinoic acid)	Colon cancer	Promotes the overexpression of XAF1.	(168)
CD147	AC-73	HCC	Inhibits dimerization of CD147.	(169)
CD133 and CD44	TX-402 (Tirapazamine)	Ovarian cancer	Decreases CD133 and CD44 levels.	(170)
HIF-1α	Noscapine	Ovarian cancer	Promotes proteasome-mediated degradation of HIF-1 α.	(171)
EphA2	4a Compound	Glioblastoma	Inhibits EphA2 directly.	(172)
VE-Cadherin	Sunitinib	Renal cell carcinoma	Inhibits Tyrosine-kinases (including VE-Cadherin).	(173)

*The different molecules with a potential VM-therapeutic effect that has been tested in different types of cancer*.

*VM, vasculogenic mimicry; HCC, hepatocellular carcinoma*.

Studies using pancreatic cancer cells showed that Ginsenoside Rg3, a tetracyclic triterpenoid saponin, reduces VM in xenograft mice models. Moreover, the expression of VE-cadherin, EphA2, MMP-2, and MMP-9 was also down-regulated after the treatment (174). Ginsenoside Rg3 has been proved in ovarian cancer derived cells restraining HIF-1α expression by activating the ubiquitin-proteasome pathway. This effect efficiently blocked migration and EMT in *in vitro* and *in vivo* ovarian cancer models, promising a novel anti-VM therapeutic agent (175, 176).

It has been shown that PARP inhibition sensitizes for chemo and radiotherapy in different types of tumors. In melanoma cells that were treated with PARP inhibitors (PJ-34, Isoquinolinone, or Olaparib) a reduction of pro-metastatic and VM markers was observed (177). PARP I inhibitors, such as Olaparib and Rupaparib, have been approved for the treatment of recurrent BRCA-associated ovarian cancer by the Food and Drug Administration (FDA); while Niraparib is used as maintenance therapy following chemotherapy for recurrent ovarian cancer (178). Nevertheless, to date there is no information about their effect on VM in ovarian cancer.

Thalidomide is an immunomodulatory agent with strong anti-angiogenic properties and has been proved in ovarian cancer, glioblastoma, hepatocellular carcinoma, and multiple myeloma in diverse clinical trials. Induction therapy with thalidomide significantly improved the overall response rate, progression free survival and overall survival (179). Previously, it has been shown that thalidomide suppresses tumor growth and angiogenesis in murine models (180). Interestingly, in a xenograft mouse model of melanoma, it was observed that mice treated with Thalidomide induced necrosis in melanoma cells. In addition, VM and tumor growth were significantly reduced compared to non-treated specimens. This effect could be related to the down-regulation of NF-kappaB signaling pathway (181). However, further studies are required to elucidate this statement.

A monoclonal antibody has been developed to target VM, unfortunately it has not been introduced for ovarian cancer treatment. This antibody targets the outer-membrane immunoglobulin-like domains of VE-cadherin, blocking receptor function. In lung cancer cells, it was observed that this antibody functions as an anti-VM agent for cancer treatment, since it inhibited the activation of the VE-cadherin-related pathway in VM (182). Due to the advantages that monoclonal antibody therapies imply, its application in ovarian cancer as an anti-VM agent is promising.

Other molecules implicated in VM in ovarian cancer, such as miR-200a, miR-27b, and miR-765 represent potential candidates for anti-tumoral therapies (85, 126, 128). Importantly, the current strategies are focused in the reduction of cancer through restoring the expression of down-regulated miRNAs, also known as miRNA replacement therapy. There are several ways to harness miRNAs in cancer cells for therapeutic purposes, including introduction of synthetic miRNA mimics, miRNA expressing plasmids, and small molecules that epigenetically alter endogenous expression of miRNAs (183). Such anti-VM strategies could represent an opportunity to venture into the study of new molecules for therapeutic purposes in ovarian cancer. Further studies will be required to prove the effectiveness of such molecules for treatment purposes.

## Concluding Remarks

Ovarian cancer is a common gynecological cancer and it is usually diagnosed in advanced stages where therapeutic success is limited. This type of tumors exhibit an aggressive phenotype characterized by a high rate of metastasis, invasion, and poor treatment response. These features are highly associated with the development of neovasculature formed by both endothelial and tumor cells. Particularly, MV is a process that may be influencing ovarian cancer poor prognosis and limited efficacy of anti-angiogenic strategies. Nevertheless, the mechanisms underlying VM formation in ovarian cancer remains unclear and deserves further studies. Recently, molecules that regulate cellular adhesion, hypoxia and EMT have been identified as key regulators of VM. Additionally, it has been shown an important post-transcriptional regulation mediated by microRNAs, that impact on the expression of VM-related proteins such as VE-cadherin, EphA2, and VEGF. Furthermore, this information has allowed the development of strategies with therapeutic potential directed against VM formation. However, subsequent studies will be necessary to elucidate the mechanisms that allow the development of conventional anti-angiogenic therapies combined with the novel anti-VM targets that improve the clinical outcomes of ovarian cancer patients.

## Author Contributions

LA-D, LO-N, JM-B, AC-P, JM-M, IM-R, and ML performed the bibliographic review, wrote, and critically revised the manuscript. ML conceived and directed the manuscript.

### Conflict of Interest

The authors declare that the research was conducted in the absence of any commercial or financial relationships that could be construed as a potential conflict of interest.
